# A novel mucopolysaccharidosis type II mouse model with an iduronate-2-sulfatase-P88L mutation

**DOI:** 10.1038/s41598-023-34541-w

**Published:** 2023-05-15

**Authors:** Ryuichi Mashima, Mari Ohira, Torayuki Okuyama, Masafumi Onodera, Shuji Takada

**Affiliations:** 1 Department of Clinical Laboratory Medicine, National Center for Child Health and Development, 2-10-1 Okura, Setagaya-ku, Tokyo, 157-8535 Japan; 2 Department of Pediatrics and Clinical Genomics, Faculty of Medicine, Saitama Medical University, Moroyama, Saitama 350-0495 Japan; 3 Department of Human Genetics, National Research Institute for Child Health and Development, 2-10-1, Okura, Setagaya-ku, Tokyo, 157-8535 Japan; 4 Department of Systems BioMedicine, National Research Institute for Child Health and Development, 2-10-1, Okura, Setagaya-ku, Tokyo, 157-8535 Japan

**Keywords:** Medical genetics, Biochemistry

## Abstract

Mucopolysaccharidosis type II (MPS II) is a lysosomal storage disorder characterized by an accumulation of glycosaminoglycans (GAGs), including heparan sulfate, in the body. Major manifestations involve the central nerve system (CNS), skeletal deformation, and visceral manifestations. About 30% of MPS II is linked with an attenuated type of disease subtype with visceral involvement. In contrast, 70% of MPS II is associated with a severe type of disease subtype with CNS manifestations that are caused by the human iduronate-2-sulfatase (*IDS*)-Pro86Leu (P86L) mutation, a common missense mutation in MPS II. In this study, we reported a novel *Ids*-P88L MPS II mouse model, an analogous mutation to human *IDS*-P86L. In this mouse model, a significant impairment of IDS enzyme activity in the blood with a short lifespan was observed. Consistently, the IDS enzyme activity of the body, as assessed in the liver, kidney, spleen, lung, and heart, was significantly impaired. Conversely, the level of GAG was elevated in the body. A putative biomarker with unestablished nature termed UA-HNAc(1S) (late retention time), one of two UA-HNAc(1S) species with late retention time on reversed-phase separation,is a recently reported MPS II-specific biomarker derived from heparan sulfate with uncharacterized mechanism. Thus, we asked whether this biomarker might be elevated in our mouse model. We found a significant accumulation of this biomarker in the liver, suggesting that hepatic formation could be predominant. Finally, to examine whether gene therapy could enhance IDS enzyme activity in this model, the efficacy of the nuclease-mediated genome correction system was tested. We found a marginal elevation of IDS enzyme activity in the treated group, raising the possibility that the effect of gene correction could be assessed in this mouse model. In conclusion, we established a novel *Ids*-P88L MPS II mouse model that consistently recapitulates the previously reported phenotype in several mouse models.

## Introduction

Mucopolysaccharidosis type II (MPS II, OMIM #309900), or Hunter syndrome, is caused by a pathogenic mutation of iduronate-2-sulfatase (IDS), characterized by an accumulation of glycosaminoglycans (GAGs), including heparan sulfate and dermatan sulfate^1^. MPS II is associated with several clinical manifestations involving the central nerve system (CNS), skeletal deformity, and visceral manifestations^2^. The prevalence of MPS II may vary depending on the population. Previous studies have reported a high prevalence in Asian countries, such as Japan, China, Taiwan, and Korea, while there is a lower prevalence among Caucasians and African Americans^3^. Enzyme replacement therapy (ERT) is an established treatment strategy that is effective for visceral disorders^4^. To improve the CNS phenotype, a lot of effort has been made in the development of therapy^5^. Such strategies have included the modification of therapeutic IDS enzymes; in this case, an IDS enzyme fused to a monoclonal antibody against an insulin receptor that is expressed in brain endothelial cells at a higher concentration^6^. Another approach involves the intrathecal administration of recombinant enzymes in the brain^7^.

There are two disease subtypes of MPS II^8^. The severe type of MPS II involves cognitive impairment and developmental delays in the body. The affected individuals typically represent maximal development at 4–6 years of age, followed by mental decline^9^. In contrast, the attenuated type of MPS II shows a milder phenotype, including visceral manifestations and bone deformation. Approximately 70% of MPS II cases are known to be severe, while the rest are linked to the attenuated type^8^. The most frequently observed genotype for the severe type of MPS II involves the recombination of *IDS-IDS2*, where the *IDS2* is a pseudogene of the IDS gene on the X chromosome. In sharp contrast, missense mutations have been commonly identified in the attenuated type of MPS II. In fact, nearly 80% of genotypes identified in this disease subtype involve missense mutations^8^. The rest of missense mutations are linked to the severe type of MPS II. Importantly, any missense mutations close to the catalytic center human Cys84 or mouse Cys86 tend to be involved in the severe type. For example, human Pro86 is a hot-spot missense mutation that is associated with the severe type^8^.

Growing evidence has demonstrated that there is an MPS disease type-specific biomarker in humans^10–12^. Among these species, a specific accumulation of UA-HNAc (1S) (where UA, HNAc, and S represent uronic acid, *N*-acetylhexosamine, and sulfur, respectively), with late retention time on reversed-phase liquid chromatography-tandem mass spectrometry (LC–MS/MS) has been described in the urine of MPS II-affected individuals^12^. This observation was further supported by the fact that this biomarker was also elevated in dried blood spots (DBSs) in neonates^13^. It is not clear whether this heparan sulfate-derived biomarker for MPS II in humans could also be elevated in an MPS II mouse model.

More recently, the three-dimensional structure of human IDS proteins has been reported^14^. Based on this model, the missense mutation involved in the attenuated type of MPS II is mostly located on the surface of the IDS protein. In contrast, those involved in the severe type were localized, at least in part, near the catalytic center human *IDS*-Cys84. The human IDS enzyme needs to be activated by the formation of a formylglycine from human *IDS*-C84 by an enzyme formylglycine generating enzyme (FGE, OMIM #607939), a Golgi enzyme encoded by the SUMF1 gene^15^. This cysteine is located within the established target motif of the FGE reaction with an amino acid sequence CXPXR, where C, X, P, and R stands for cysteine, any amino acids, proline, and arginine, respectively. In this study, we generated a novel *Ids*-Pro88Leu (P88L) MPS II mouse model harboring an analogous pathogenic mutation of human *IDS*-P86L (ClinVar accession: VCV000527322.5; variation ID: 527322) and reported its biochemical phenotype.

## Results

### Generation of a novel *Ids*-P88L MPS II mouse model

**Figure 1:**
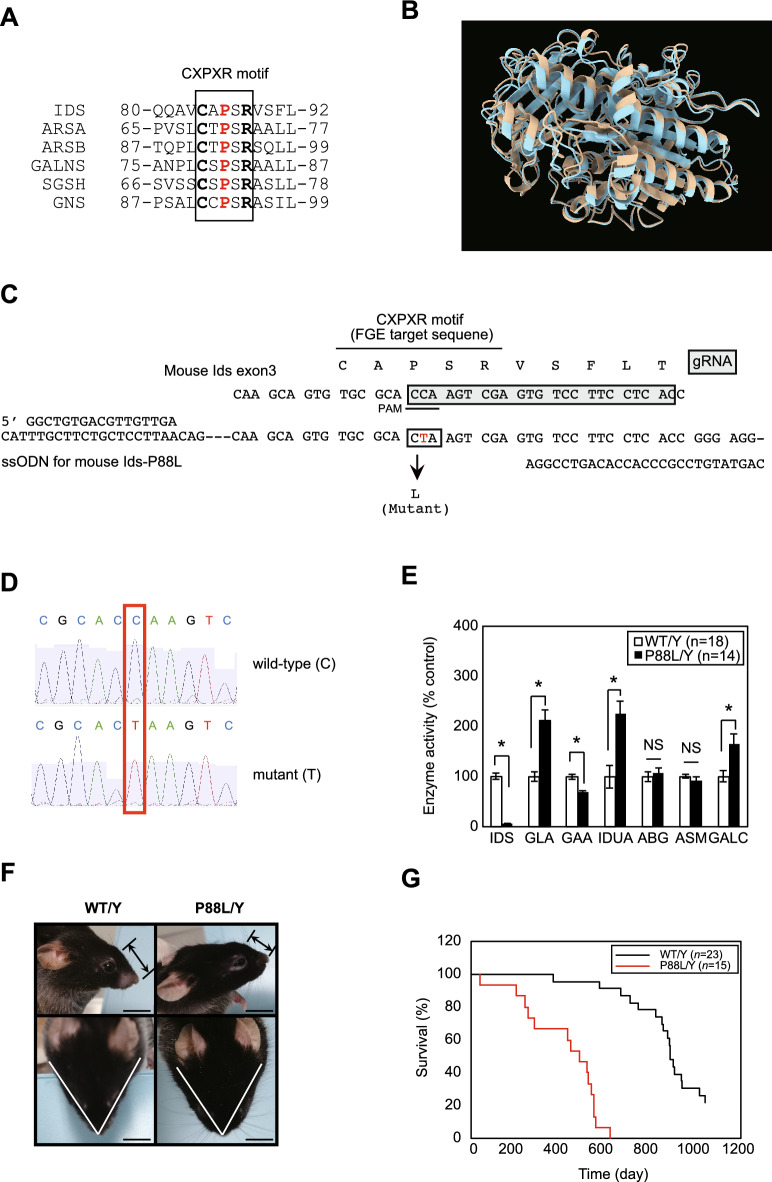
Generation of a novel *Ids*-P88L MPS II mouse model. (**A**) Sequence alignment of CXPXR motif in human LSD sulfatases. (**B**) 3D structure of human IDS wild-type (beige) and P86L (blue). Each prediction structure was calculated based on amino acid sequence of human IDS (UniProt P22304) by AlphaFold2 (https://colab.research.google.com/github/sokrypton/ColabFold/blob/main/AlphaFold2.ipynb). Superimposition of 3D structures were performed using ChimeraX software (https://www.cgl.ucsf.edu/chimerax/). (**C**) Structure of the mouse Ids genome. A replacement of Pro with Leu was induced by gRNA, as shown in the gray box. The PAM sequence was underlined. The CXPXR motif, an FGE target sequence, was marked using a bar. (**D**) Electropherogram of a BigDye-labeled PCR amplicon of the genome prepared from a wild-type (top, C) and mutant mouse (bottom, T). (**E**) Enzyme activity of IDS and other LSD enzymes in DBS. (**F**) Gross appearance of *Ids*-P88L MPS II mouse model. A shorten nasal bone length (upper) and a widen facial appearance (lower) of *Ids*-P88L MPS II mouse model was presented. A bar indicates 1 cm. (**F**) Survival curve of a novel *Ids*-P88L MPS II mouse model.

CXPXR motif is a target motif in IDS protein for FGE (Fig. 1A,B, Supplementary Fig. S1). To generate a novel *Ids*-P88L MPS II mouse model, a guide RNA (gRNA) was microinjected into the nucleus and cytoplasm of in vitro fertilized oocytes, and they were transferred to a pseudopregnant mouse (Fig. 1C). Pups were identified using DNA sequencing (Fig. 1D). We were able to obtain six pups of the expected mouse *Ids*-P88L (c.C263T) mutants, equivalent to human *IDS*-P86L (c.C257T) (Table 1). These mice were born at the Mendelian ratio, as expected, based on the genotyping using an allele-specific quantitative PCR (Supplementary Fig. S2). The IDS enzyme activity of this mouse model was impaired in DBSs, whereas other enzyme activity of lysosomal storage disorder (LSD), such as α-galactosidase A (GLA), α-glucosidase (GAA), α-iduronidase (IDUA), acid β-glucosidase (ABG), acid sphingomyelinase (ASM), and galactosylceramidase (GALC), either remained within normal range or elevated significantly by LC–MS/MS-based enzyme assay (Fig. 1E, Supplementary Table S1). The established *Ids*-P88L MPS II mouse model exhibited an abnormal facial appearance with a short lifespan (Fig. 1F,G).

**Table 1:** Summary of genomic alteration of F0 mice induced by genome editing.

Genomic alteration	Sequence							Male	Female	Total
	CCA	AGT	CGA	GTG	TCC	TTC	CT			
C-to-T conversion	CtA	AGT	CGA	GTG	TCC	TTC	CT	5	3	8
A T-insertion	CCA	AGTtCGA		GTG	TCC	TTC	CT	1	2	3
A G-insertion	CCA	AGTgCGA		GTG	TCC	TTC	CT	1	0	1
5-Nucleotide deletion	CCA	– – – – – – A	GTG	GTG	TCC	TTC	CT	0	2	2
Small deletion^a^ and others	N/A							2	4	6
Sum								9	11	20

^a^The detail of genomic alteration will be described elsewhere.

### Visceral phenotype of the novel Ids-P88L MPS II mouse model

**Figure 2:**
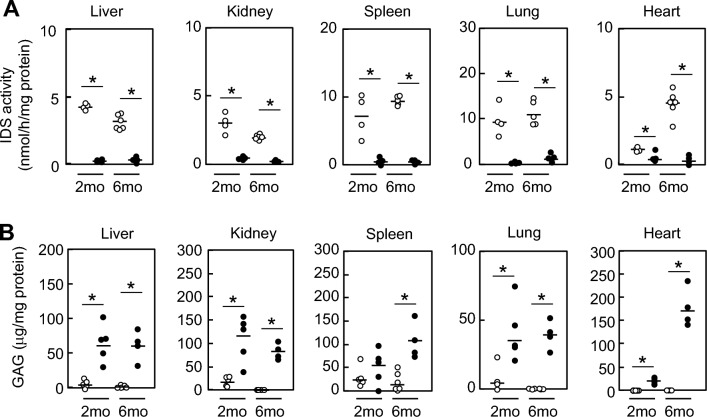
Visceral phenotype of a novel *Ids*-P88L MPS II mouse model. (**A**) The GAG levels in the visceral organs. The concentration of GAG in the liver, kidney, spleen, lung, and heart was quantified using the 1,9-dimethylmethylene blue-based method (Blyscan, UK). Animals were examined at ages 2 and 6 months. Open circle: wild-type; closed circle: a novel *Ids*-P88L MPS II mouse model. Each circle represents an individual mouse (*n* = 4—6). (**B**) Enzyme activity of IDS in visceral organs. Tissues were harvested as described, and IDS activity was determined using LC–MS/MS as described. An aliquot of tissue was reacted with an enzyme substrate for IDS at 37 °C for 20 h. The reaction product was then extracted in ethyl acetate and methanol, dried, and reconstituted using a starting solution of LC–MS/MS assay. The level of enzyme activity was quantified using a deuterated internal standard, as described in the Materials and Methods section. Each circle represents an individual mouse (*n* = 3–6). Open circle, wild-type, closed circle, *Ids*-P88L MPS II mouse model. A bar indicates the mean value. * denotes *P* < 0.05.

We next investigated the visceral phenotype of our newly established Ids-P88L MPS II mouse model. First, we found that the IDS enzyme activity in the liver, kidney, spleen, lung, and heart was significantly decreased at 2 and 6 months of age (Fig. 2A). We then examined the level of GAGs, a well-established biomarker for all MPS disease types, including MPS II. As observed in humans, the GAG concentration in the live, kidney, spleen, lung, and heart was elevated in this mouse model at 2 and 6 months of age, respectively (Fig. 2B).

### An accumulation of UA-HNAc (1S) (late retention time) in the novel Ids-P88L MPS II mouse model

**Figure 3:**
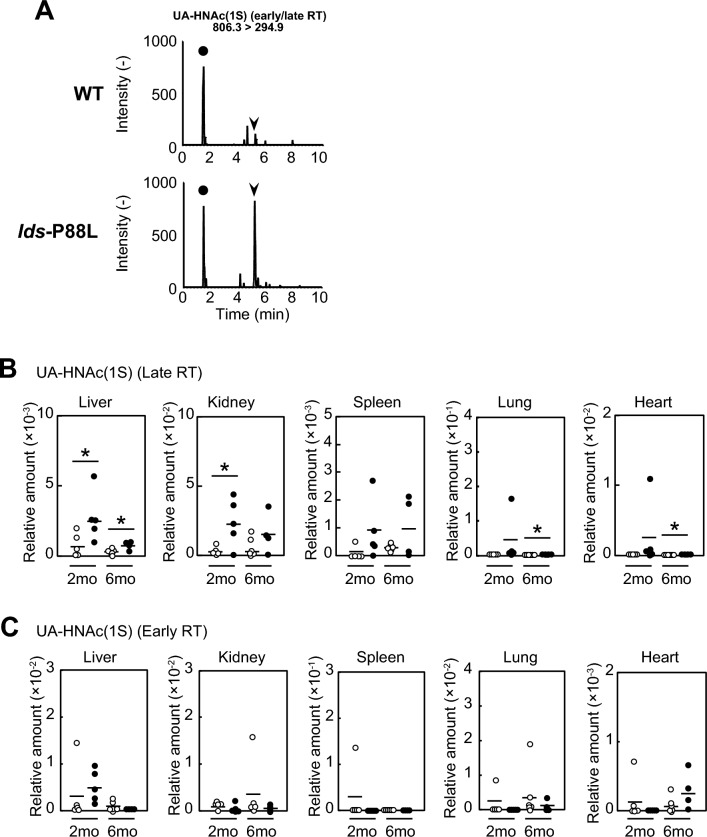
Quantification of MPS disease-specific biomarkers in visceral organs of a novel *Ids*-P88L MPS II mouse model. (**A**) Representative chromatograms for UA-HNAc (1S) (early and late retention times). The biomarkers, together with an internal standard, in tissue homogenate were derivatized using PMP reagent at 70 °C for 90 min, followed by extraction using chloroform. The dried sample was reconstituted with a starting solution for LC–MS/MS assay. A negative mode of the electrospray ionization method was used for quantification (Supplementary Table S2). An individual peak of UA-HNAc (1S) migrating at a late retention time (5.1 min) and an early retention time (1.8 min) was indicated by an arrowhead and a closed circle in the chromatogram, respectively. Top, wild-type; bottom, *Ids*-P88L MPS II mouse model. (**B**) Quantitative results of UA-HNAc (1S) (late retention time). (**C**) Quantitative results of UA-HNAc (1S) (early retention time). The data were expressed as the relative amount after normalization using protein concentration (*n* = 4–6). Each circle represents an individual mouse. Open circle, wild-type, closed circle, Ids-P88L MPS II mouse model. A bar indicates the mean value. *P < 0.05 (**D**).

Heparan sulfate is one of the molecular species of GAGs. UA-HNAc (1S) (late retention time) is a recently reported MPS II-specific biomarker from heparan sulfate^13^. Thus, we further examined the levels of putative biomarkers, including UA-HNAc (1S) (late retention time), in our Ids-P88L MPS II mouse model (Fig. 3, Supplementary Table S2). We found an elevation of UA-HNAc (1S) (late retention time) in the liver of this model at 2 and 6 months of age, respectively (Fig. 3A arrowhead and Fig. 3B). An occasional elevation of UA-HNAc (1S) (late retention time) was also observed in other tissues, such as the kidney, at 2 months, and the lung and heart, at 6 months, respectively. In contrast, another biomarker, such as UA-HNAc (1S) (early retention time), a biomarker for MPS I, was not consistently elevated at 2 and 6 months of age in our Ids-P88L MPS II mouse model (Fig. 3C). Other biomarkers for different MPS disease type, such as HN-UA (1S) for MPS IIIA and HNAc-UA (1S) for MPS IVA, were also not elevated in this model (Supplementary Fig. S3).

### Therapeutic effect of nuclease-mediated gene editing

**Figure 4:**
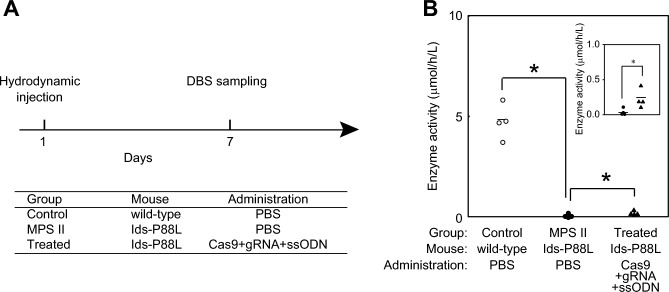
The effect of nuclease-mediated correction of IDS enzyme activity. (**A**) Timeline of the experimental protocol. Prior to the experiments, the mice were weighed and randomized based on body weight. The mice were grouped as (1) a control group with wild-type mice treated with PBS; (2) an MPS II disease group with *Ids*-P88L MPS II mouse model treated with PBS; and (3) a treated group with an *Ids*-P88L MPS II mouse model treated with a mixture of Cas9 nuclease, gRNA, and ssODN for template DNA. At day 7, the blood was collected by retro-orbital plexus and spotted onto filter paper, dried at room temperature overnight, and stored at -20 °C prior to use. (**B**) The IDS enzyme activity of the nuclease-treated *Ids*-P88L MPS II mouse model. The enzyme activity was quantified using LC–MS/MS as described. Open circle, control group (*n* = 4); closed circle, *Ids*-P88L MPS II mouse model (*n* = 4); closed triangle, treated group (*n* = 4). A bar indicates the mean value. *P < 0.05. *Inset,* enzyme activity of Ids-P88L MPS II mouse model treated with PBS and nuclease-were magnified.

Finally, we examined the therapeutic effect of gene editing on this *Ids*-P88L MPS II mouse model. We first prepared three groups of mice: (1) a control group with wild-type mice treated with phosphate-buffered saline (PBS); (2) an MPS II disease group with an *Ids*-P88L MPS II mouse model treated with PBS; and (3) a treated group with an *Ids*-P88L MPS II mouse model treated with a mixture of Cas9 nuclease, gRNA, and single strand oligodeoxynucleotide (ssODN) for template DNA (Fig. 4A, Supplementary Fig. 4). At the time of injection, the mice were treated with either PBS or a therapeutic mixture via hydrodynamic injection. At day 7, the blood was collected, and the IDS enzyme activity was measured. We found that, in the control group, the IDS enzyme activity in DBS was 4.72 ± 0.44 μmol/h/L blood (mean ± SEM, *n* = 4) (Fig. 4B). Under the same conditions, the IDS enzyme activity of the MPS II disease group was 0.04 ± 0.03 μmol/h/L blood (*n* = 4). When we treated the *Ids*-P88L mouse model with a therapeutic mixture, we found that these mice showed 0.23 ± 0.07 μmol/h/L blood (*n* = 4) of IDS enzyme activity. Under these conditions, the elevation of IDS enzyme activity in the treated group was calculated as 4.2% based on the measured IDS enzyme activity.

## Discussion

In this study, we established a novel Ids-P88L MPS II mouse model. The phenotype of this model recapitulated the previously established disease model, such as attenuated enzyme activity and an elevation of GAG in the body. These data indicate that our animal model exhibited a similar phenotype as previously reported^16–18^. Furthermore, UA-HNAc (1S) (late retention time) is a recently reported novel biomarker for MPS II in humans^12^. We also ensured that this biomarker was elevated in our MPS II mouse model. The effect of nuclease-mediated gene correction in the *Ids*-P88L MPS II mouse model appeared to be marginal, raising the possibility that an alternative strategy, including the expression of therapeutic *IDS* cDNA, could provide a better therapeutic outcome.

There are two roles for human *IDS*-P86 and mouse *Ids*-P88. First, matured sulfatase, including IDS, requires formylglycine, an oxidized form of cysteine, by an enzyme called FGE^15^. These cysteine residues are found in the CXPXR motif, a known short sequence specifically found in all sulfatases in humans and mice. In fact, this motif has been identified in 87 proteins in humans by bioinformatics research^19^, whereas there are only 17 sulfatase genes in humans^15^, suggesting that P in the CXPXR motif is a prerequisite for sulfatase activity (Fig. 1A,B). In fact, a previous biochemical study using a 23-mer peptide demonstrated that FGE reacts with IDS at 30% of the reaction rate compared to arylsulfatase A/cerebroside sulfatase, demonstrating that the IDS enzyme is a reasonably good substrate for FGE^20^. Second, empirical evidence has suggested that because proline is a naturally occurring imino acid lacking a Cα atom that is capable of freely rotating α-amino acid in its polypeptide, the replacement of proline with other natural amino acids (i.e. α-amino acid) leads to the deformation of the protein’s tertiary structure. Such a hypothesis could also be applicable in human *IDS*-P86L because this mutation leads to a severe type of MPS II disease subtype with CNS involvement.

Genome editing technology is now a widely used DNA manipulation technique that has revolutionized experimental biology^21^. Essentially, a Cas9 protein binds to the target DNA sequence together with a gRNA through a short sequence with three nucleotides known as a protospacer adjacent motif (PAM) sequence, followed by DNA cleavage by Cas9 nuclease. There are two mechanisms by which DNA cleavage occurs. One is non-homologous end joining (NHEJ), which occurs regardless of cell cycle status. In contrast, homology-directed repair (HDR) is another mechanism that is active only during the G2/S phase. When gRNA was injected into mouse fertilized egg cells, 65% of a C-to-T conversion (5 males and 8 females in 20 mice) was detected in our experiment (Table 1). Other mutations included two 1-base insertions, a 5-base deletion, and a small deletion, suggesting that NHEJ appeared to be predominant. Genome editing technology has also been receiving a lot of attention as a therapy for genetic disorders^22^. In this sense, HDR provides a platform for therapeutic strategies by introducing a correct nucleotide sequence. Currently, gene correction by HDR could occur at low frequency, as reported in previous studies^23–25^. In fact, our results, with a marginal correction of enzyme activity, appear to be consistent with these data.

Gene therapy is an anticipating therapy. For example, adeno-associated virus (AAV)-based intravenous infusion is one such strategy of gene therapy in humans^26^. To gain its maximal effect, the AAV-based transgene has been administered multiple times to the site of disease or a relatively small compartment, such as the brain^27,28^. Currently, in addition to gene therapy, a variety of treatment strategies have been developed. ERT is an established therapy that infuses a recombinant therapeutic enzyme into the patient. A classical delivery method infuses a recombinant enzyme intravenously^4^. This therapy is effective for visceral manifestations, including hepatosplenomegaly. In sharp contrast, this conventional ERT is not effective for CNS manifestation. To overcome this drawback, a recombinant IDS enzyme fused to an anti-insulin receptor antibody has been developed^5,6,18^. Separately, intrathecal administration of the IDS enzyme has also been under development and is in the late stages of clinical studies^7,29^. In the case of MPS II, a hematopoietic stem cell transplant (HSCT) appears not to be chosen for therapy in clinical settings, while other MPS disease types, such as MPS I, IIIA-D, IVA/B, VI, and VII, are treated with HSCT. The reason for this difference between MPS II and other types of MPS remains unclear, but it has been suggested that hematopoietic-cell-based cross-correction may not be efficient in the IDS enzyme.

Heparan sulfate is degraded in the lysosome with multiple enzymes, including IDS^30^. The biochemical nature of UA-HNAc (1S) (late retention time) has not yet been elucidated, but this biomarker is highly likely to contain a disaccharide consisting of uronic acid and *N*-acetylhexosamine with one sulfate group^11,12^. Because humans and mice have many hydrolases, the responsible enzyme has not yet been identified^31^. Given that non-specific hydrolysis of heparan sulfate into this biomarker would play a major role, the identification of such hydrolase might be rather difficult because (1) these hydrolases are likely to present at high concentrations in the cells and (2) the concentration of heparan sulfate-derived degradation products is much lower than endogenous or exogenous disaccharides, such as lactose, maltose, and sucrose, particularly in the liver. Apart from humans and mice, recent studies have shown that several metabolizing enzymes for heparan sulfate are also found in *Bacteroides* and *Firmicutes* intestinal bacteria^32–34^. The mechanism of its degradation in the bacterium appears to be different from that of the mammalian mechanism. In bacteria, heparin lyase 1–3 play an important role at the early stage of heparan sulfate degradation. From the viewpoint of substrate specificity, heparin lyase 1 catalyzes the hydrolysis of glycosidic linkage of the non-reducing end of iduronic acid, while heparin lyase 3 degrades heparan sulfate next to glucuronic acid^35^. Aside from them, heparan lyase 2 has both activity for heparan sulfate degradation. Furthermore, it is known that certain intestinal bacteria, such as *Bacteroides iotathetaomicron*, contain a variety of additional heparan sulfate-modifying enzymes^32–34,36,37^. However, an estimated contribution of gut microbiome-derived enzymes has been suggested at approximately 10% in healthy humans^38,39^, thus their contribution to an elevation of HS disaccharide species seemed to be small.

In conclusion, we established a novel *Ids*-P88L MPS II mouse model. Overall, the phenotype of this animal model appeared to be consistent with previously established MPS II mouse models. Future studies on the development of therapeutic strategies could be performed using this animal model.

## Materials and methods

### Reagents

1-Phenyl-3-methyl-5-pyrazolone (PMP: CAS 89-25-8) was purchased from Tokyo Chemical Industries (Tokyo, Japan). Chondroitin disaccharide di-4S (∆HexA-GalNAc(4S), CAS 136144-56-4) was purchased from Carbosynth (Berkshire, UK). The other reagents were of the highest grade and commercially available.

### Generation of the *Ids*-P88L MPS II mouse model

For the generation of mutant mice, a gRNA with the following sequence was used: GTGAGGAAGGACACTCGACACTCGACTTGG, where PAM was underlined^21^. The sgRNA was synthesized using a CUGA7 gRNA synthesis kit (Nippon Gene Co., Ltd., Toyama, Japan) according to the manufacturer’s instructions. A mixture of Cas9 protein (100 ng/μL), sgRNA (250 ng/μL), and ssODN (100 ng/μL) was microinjected into the nucleus and cytoplasm of in vitro fertilized oocytes prepared from C57BL/6 × DBA/2 F1 hybrid. The oocytes were cultured overnight and transferred to the oviducts of pseudopregnant ICR females. The pups were identified using DNA sequencing, as described below.

### Animals

The F0-generation mice were mated with C57BL/6 mice (CLEA Japan, Tokyo, Japan). The genotype of the F1 mice was confirmed using the Sanger sequence. The F1 mice were intercrossed, and their offspring F2 animals were used for further experiments. The mice were fed with standard chow ad libitum (CE-2, CLEA Japan, Tokyo, Japan) and were maintained on 12-h-light/dark cycles (8:00–20:00). This study is reported in accordance with ARRIVE guidelines (https://arriveguidelines.org).

### Animal ethics

The protocol for animal experiments was approved by the Animal Committee of the National Center for Child Health and Development, Tokyo, Japan. All methods were carried out in accordance with relevant guidelines and regulations.

### Genotyping

For genotyping, genomic DNA from mouse tail clippings or tissue was isolated by standard procedures. For DNA sequencing-based genotyping, the genome DNA was amplified using a set of the following primers: forward primer, GGCAAGGCCCTAATCCTACT; reverse primer, AGAAACAAAAGGCCCAGGTT. The PCR product was then diluted using distilled water, treated with ExoSAP-IT and labeled with a BigDye terminator sequencing kit (Thermo Fischer Scientific, Waltham, MA). Finally, the fluorescence-labeled DNA was sequenced using a capillary sequencer of 3130xl (Thermo Fischer Scientific). For probe-based genotyping, we used the following primers: forward: CATTTGCTTCTGCTCCTTAAC; reverse: CAATTCCTACTGGAGGGTACA; Fluorescein-aminohexyl (FAM)-labeled probe for wild-type allele: TCG + ACT + T + G + GTGC; Hexachlorofluorescein (HEX)-labeled probe for mouse Ids-P88L allele: TCGA + CT + T + A + GTGCG, where + denotes locked DNA (Integrated DNA technologies, Tokyo, Japan). Genotyping was performed using a Thunderbird Probe qPCR mix (Toyobo, Tokyo, Japan). The temperature was maintained at 95 °C for 10 min, followed by 40 cycles at 95 °C for 15 s and 60 °C for 15 s. The QuantStudio 12 K Flex Real-Time PCR System was used (Thermo Fischer Scientific).

### DBS preparation

Blood was collected from the retro-orbital vein with a 75-μL plain hematocrit capillary (Hirschmann, Eberstadt, Germany). Blood was spotted on filter paper for newborn screening (Advantec, Tokyo, Japan)^40^. The samples were dried at room temperature overnight and then stored at − 20 °C prior to use.

### GAG quantification

Tissue homogenate was pretreated with Proteinase K (0.2 mg/mL, Sigma-Aldrich, St Louis, MO) in 100 mM Tris–HCl (pH 8) at 56 °C overnight. Then, the amount of released GAG was quantified using a colorimetric assay with 1,9-dimehtyl-methylene blue dye (Blyscan, Northern Ireland, UK), as previously reported^41,42^. A standard curve was generated using chondroitin sulfate. The GAG level in the tissue extract was adjusted by its protein concentration, as determined by a BCA assay kit (Nacalai Tesque, Kyoto, Japan).

### Quantification of IDS enzyme activity

Liver, kidney, spleen, lung, and heart tissues were homogenized in MilliQ water (Millipore, Tokyo, Japan), and protein extracts were obtained by manual homogenization. The IDS activity in the homogenate was determined as previously described with a slight modification^43,44^. In brief, the sample (5 μL) was incubated with the substrate at 37 °C for 20 h (PerkinElmer, Waltham, MA). After the termination of the enzyme reaction with methanol and ethyl acetate, the reaction products were extracted into ethyl acetate using a 96-well plate (cat# 260252, Thermo Fischer Scientific). An aliquot (0.2 mL) of the upper layer was evaporated under a nitrogen stream. The residue was then reconstituted with a mixture of acetonitrile/water = 20/80 with 0.2% formic acid (Kanto Chemicals, Tokyo, Japan). Finally, enzyme activity was determined by quantifying the accumulation of the enzyme reaction product using LC–MS/MS equipped with a Xevo TQ-S micro tandem mass spectrometer and an H-class UPLC chromatograph (Waters Corporation, Milford, MA)^43^.

### Quantification of MPS-specific biomarkers

The MPS-specific biomarkers were quantified using LC–MS/MS as described previously^45^. In brief, an aliquot of tissue homogenate (10 μL) was mixed with a previously described PMP solution (0.1 mL) and reacted at 70 °C for 90 min for derivatization. The reaction was then acidified using 0.5 M formic acid (0.5 mL, Kanto Chemicals, Tokyo, Japan) and chloroform (0.5 mL, Wako Pure Chemicals, Tokyo, Japan). After centrifugation at 12,000 rpm for 5 min at room temperature, the lower layer was discarded. This was performed four times in total. Then, the aqueous layer was dried under nitrogen at 70 °C. Finally, the sample was reconstituted with a solution containing 0.1% formic acid in methanol/water = 10/90 (0.3 mL). The PMP derivatives were separated on a BEH C18 column (1.7 μm, 2.1 × 50 mm, Waters Corporation) at 0.3 mL/min at 40 °C. A TQ-S micro tandem mass spectrometer and an H-class UPLC chromatograph were used (Waters Corporation).

### Longevity observation

The mice were continuously observed for the development of humane endpoint criteria, or until death occurred. When the symptoms of late-stage clinical manifestation, such as urine retention, rectal prolapse, and protruding penis, became irreversible, or when the mice showed significant weight loss, dehydration, or morbidity, it was considered the humane endpoint.

### Hydrodynamic injection

At day 1, the mice (approximately 30–40 g) were injected with either 10% (v/w) PBS or PBS containing a mixture of a plasmid of Cas9 (2.5 μg/mL), a plasmid of gRNA (12.5 μg/mL), and an ssODN of template DNA (75 ng/mL) by retro-orbital vein. On day 7, blood was removed for DBS preparation by a microcapillary, as described above.

### Statistics

Data were analyzed using the Student’s *t*-test. For all comparisons, significance was set at *P* < 0.05.

## Supplementary Information

Supplementary Information 1.

Supplementary Information 2.

## Data Availability

The datasets of the current study are available from the corresponding author on reasonable request.
