# Supplementary material for: A novel mucopolysaccharidosis type II mouse model with an iduronate-2-sulfatase-P88L mutation
Source: Sci Rep. 2023 May 15;13:7865. doi: 10.1038/s41598-023-34541-w (PMC10185571; doi:10.1038/s41598-023-34541-w)
Supplement: Supplementary file 1 — Supplementary Information 1. [file 41598_2023_34541_MOESM1_ESM.pdf]

**A**

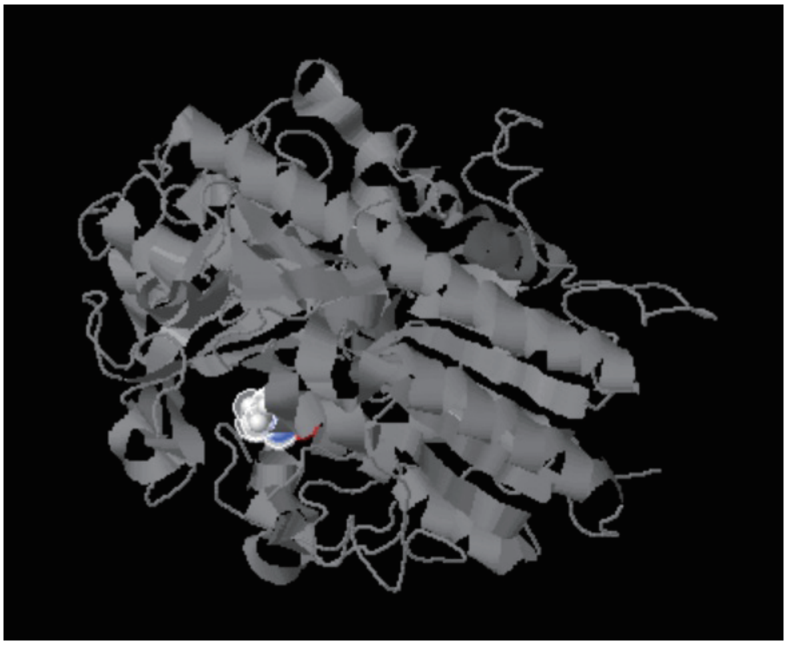

**B**

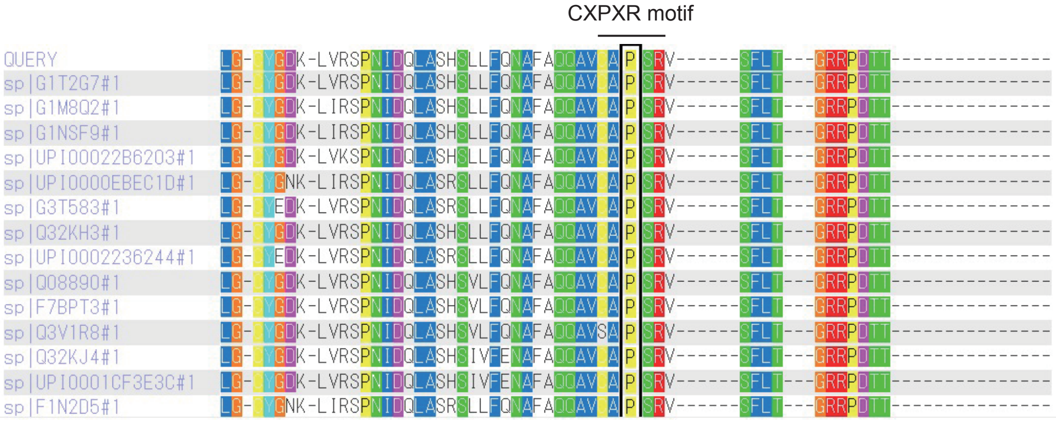


Shown are 75 amino acids surrounding the mutation position (marked with a black box)

Supplementary Fig S1
Mashima R et al.

A

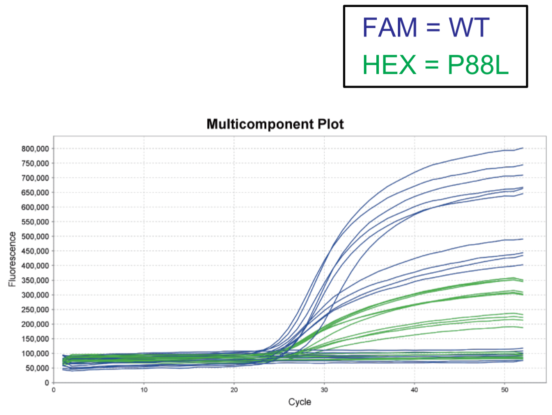


B

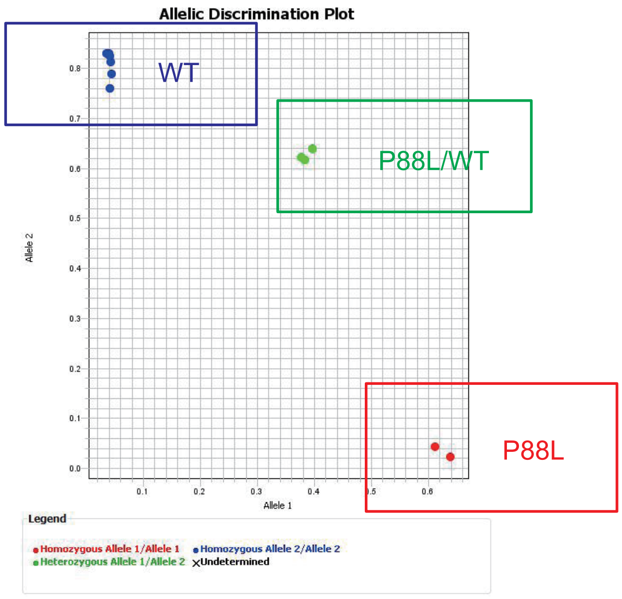


Supplementary Fig S2
Mashima R et al.

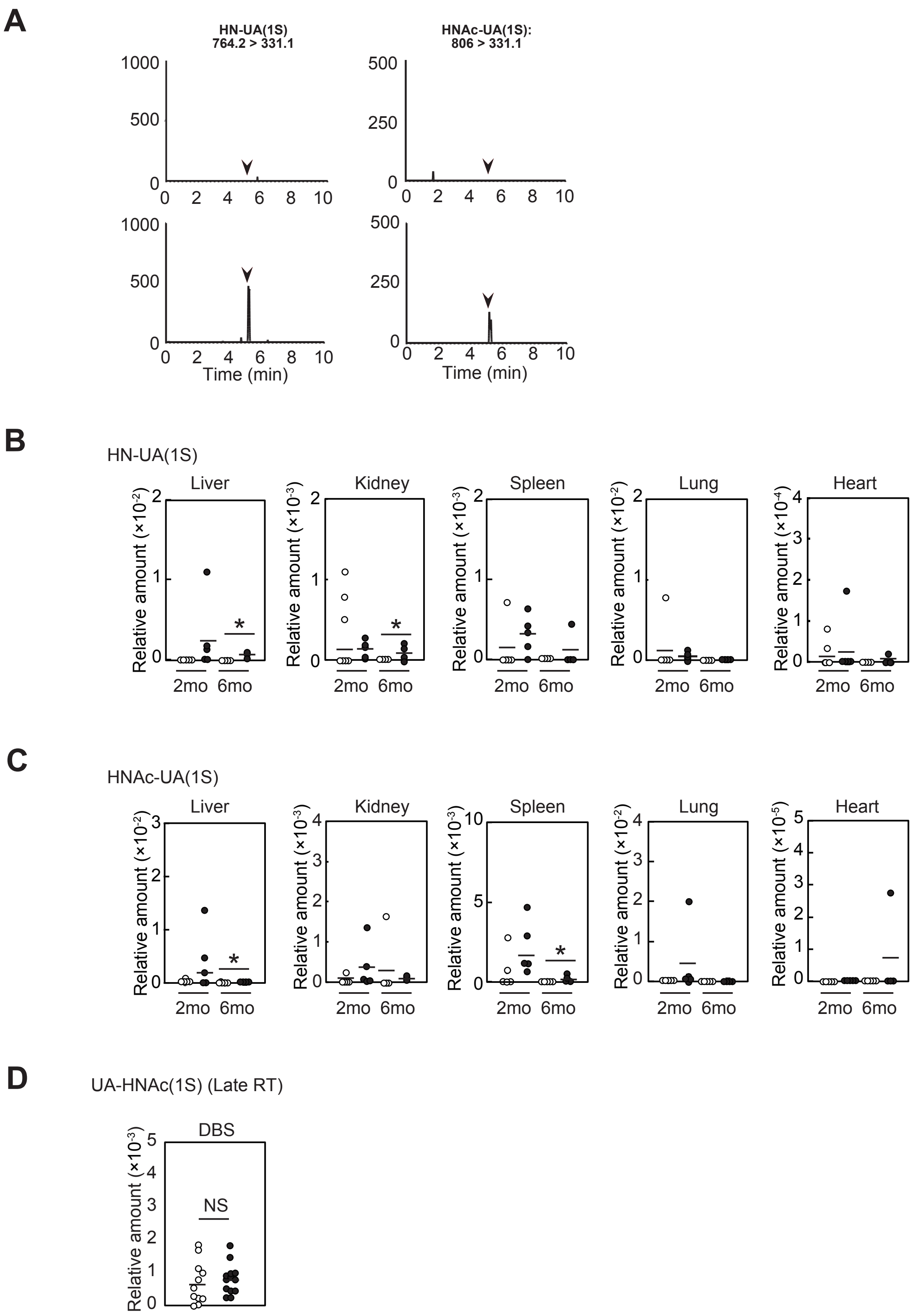


Supplementary Fig S3
Mashima R et al.

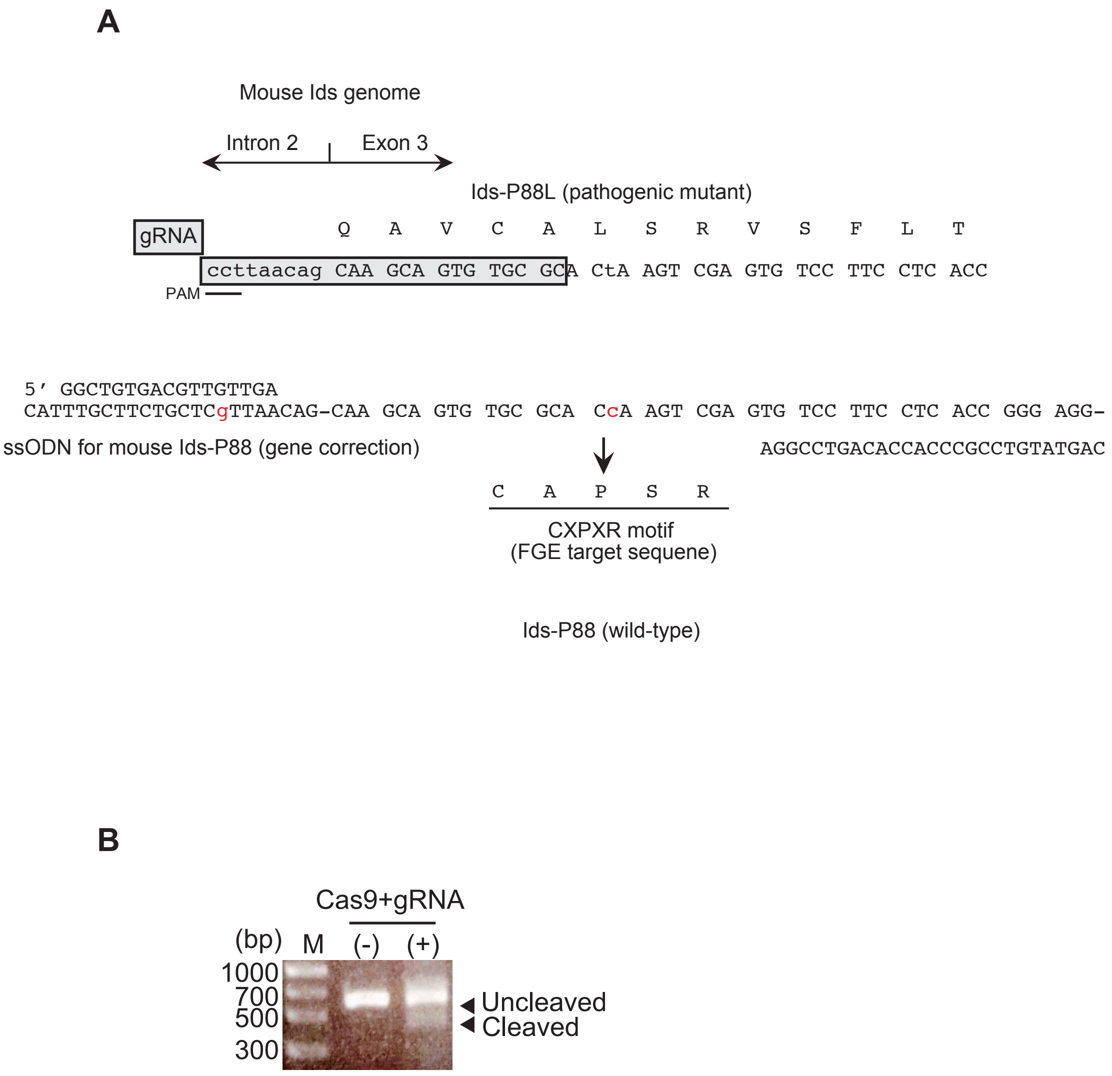


Supplementary Fig S4
Mashima R et al.
