# Supplementary material for: A novel mucopolysaccharidosis type II mouse model with an iduronate-2-sulfatase-P88L mutation
Source: Sci Rep. 2023 May 15;13:7865. doi: 10.1038/s41598-023-34541-w (PMC10185571; doi:10.1038/s41598-023-34541-w)
Supplement: Supplementary file 2 — Supplementary Information 2. [file 41598_2023_34541_MOESM2_ESM.pdf]

Supplementary Information

A novel mucopolysaccharidosis type II mouse model with an iduronate-2-sulfatase-P88L mutation

Running title: A novel *Ids*-P88L MPS II mouse model

Ryuichi Mashima,1* Mari Ohira,1 Torayuki Okuyama,1,2 Masafumi Onodera,3 Shuji Takada,4

1Department of Clinical Laboratory Medicine, National Center for Child Health and Development, 2-10-1 Okura, Setagaya-ku, Tokyo 157-8535, Japan;
2Department of Pediatrics and Clinical Genomics, Faculty of Medicine, Saitama Medical University, Moroyama, Saitama 350-0495, Japan;
3Department of Human Genetics, National Research Institute for Child Health and Development, 2-10-1, Okura, Setagaya-ku, Tokyo, 157-8535, Japan;
4Department of Systems BioMedicine, National Research Institute for Child Health and Development, 2-10-1, Okura, Setagaya-ku, Tokyo, 157-8535, Japan.

*To whom correspondence should be addressed: Ryuichi Mashima, PhD
Electronic address: mashima-r@ncchd.go.jp.
FAX: +81-3-3417-2238

Supplementary materials and methods

Preparation of sgRNA

The sgRNA was generated using CUGA7 gRNA synthesis kit according to manufacture's instruction (Nippon Gene Co., Ltd., Toyama, Japan). First, the template DNA was prepared by PCR with a combination of 0.4 μM primer 1: CTAATACGACTCACTATAGGCGCACACTGCTTGCTGTTAGTTTTAGAGCTAGAAATAGCA, 0.04 μM primer 2: AAAAGCACCGACTCGGTGCCACTTTTTCAAGTTGATAACGGACTAGCCTTATTTTAACTTGCTATTTCTAGCTCTAAAAC, and 0.4 μM primer 3: AAAAGCACCGACTCGGTGCC. The synthesized template DNA was quantified by a comparison with known amount of DNA after separation with agarose gel electrophoresis followed by ethidium bromide detection. Then, the reaction of sgRNA synthesis was performed at 37°C. Next, the template DNA was digested using DNase I. Finally, sgRNA was purified by a spin-column. The concentration of sgRNA was quantified using a spectrophotometer NanoDrop One (Thermo Fisher Scientific) as described.

In vitro digestion assay

A 615 bp DNA fragment containing the target sequence of gRNA was PCR-amplified using primer 1: CCTAACACGCTTAAGACAGGCTT and primer 2: CCTAACACGCTTAAGACAGGCTT. The known amount of DNA (100 ng) was reacted with a complex of Cas9 protein (0.5 μg, Nippon Gene Co., Ltd.) and sgRNA (50 ng) in 50 mM Tris-HCl (pH 7.9) containing 100 mM NaCl, 10 mM $MgCl_2$ and 1 mM dithiothreitol at 37°C for 2 h. The formation of hydrolyzed DNA fragments was detected using 2.5% agarose gel electrophoresis with ethidium bromide detection.

Quantification of enzyme activity for lysosomal storage disorders

Liver, kidney, spleen, lung, and heart tissues were homogenized in MilliQ water (Millipore, Tokyo, Japan), and protein extracts were obtained by manual homogenization. The enzyme activity in the homogenate was determined as previously described with a slight modification [1][2]. In brief, the sample (5 μL) was incubated with the substrate at 37°C for 20 h (PerkinElmer, Waltham, MA). After the termination of the enzyme reaction with methanol and ethyl acetate, the reaction products were extracted into ethyl acetate using a 96-well plate (cat# 260252, Thermo Fischer Scientific). An aliquot (0.2 mL) of the upper layer was evaporated under a nitrogen stream. The residue was then reconstituted with a mixture of acetonitrile/water = 20/80 with 0.2% formic acid (Kanto Chemicals, Tokyo, Japan). Finally, enzyme activity was determined by quantifying the accumulation of the enzyme reaction product using LC-MS/MS equipped with a Xevo TQ-S micro tandem mass spectrometer and an H-class UPLC chromatograph (Waters Corporation, Milford, MA).

Supplementary Table S1 LC-MS/MS conditions for MPS-specific biomarkers

LC	ACQUITY UPLC H-Class (Waters)
MS	Xevo TQ-S micro (Waters)
Column	ACQUITY BEH C18 (Waters)
	Particle diameter: 1.7 μm
	Internal diameter: 2.1 mm
	Length: 50 mm
Wash solvent	Acetonitrile
Purge solvent	5% Acetonitrile/95% water
Mobile phase A	0.1 % formic acid in 10 % methanol/90 % water
Mobile phase B	0.1 % formic acid in 90 % methanol/10 % water
Gradient (%B)	0.0-5.0 min: 15%-22.5%B
	5.0-5.5 min: 22.5%-90%B
	5.5-7.5 min: 90%B
	7.5-8.0 min: 90-15%B
	8.0-12.0 min: 15%B
Flow rate	0.3 mL/min
Injection volume	5 μL
Sample loop volume	15 μL
Autosampler temperature	10°C
Ionization mode	Electrospray ionization.
Mode	Negative

Supplementary Table S2 Mass spectrometric conditions for MPS-specific biomarkers

ID	Name of compound	Specificity[a]	Retention time (min)	ESI mode	MS1 (m/z)	MS2 (m/z)	Cone (V)	Collision (V)	Dwell time (ms)
1	HNAc(1S)	MPS IIID, MPSIVA	6.9	Negative	630.4	256.1	24	28	10
2	$(HNAc\text{-}UA)_2(2S)$	Not reported	7.0	Negative	632.3	298.0	30	34	10
3	$(HN\text{-}UA)_2\text{-}HNAc(2S)$	MPS IIIC	7.1	Negative	691.8	605.0	28	14	10
4	HN-UA(1S)	MPS IIIA	Not detected	Negative	764.2	331.1	32	26	10
5	Internal standard	Not applicable	6.9	Negative	788.1	534.1	42	24	10
6	HNAc-UA(1S)	MPS IVA	6.9	Negative	806.0	331.1	28	28	10
7	UA-HNAc(1S) (early retention time)	MPS I	1.8	Negative	806.3	294.9	40	27	10
8	UA-HNAc(1S) (late retention time)	MPS II	6.9	Negative	806.3	294.9	40	27	10
9	UA-HN-UA(1S)	Not reported	Not detected	Negative	940.0	331.1	44	34	10
10	$(HNAc\text{-}UA)_2(1S)$	MPS IIIB	Not detected	Negative	1185.2	931.4	30	32	10
11	$(Hex\text{-}HNAc)_2(2S)$	Not reported	6.8	Negative	1240.0	256.1	28	36	10

[a]Reported in Saville JT et al Genet Med 21 (2019) 753-757. HNAc: *N*-acetylhexosamine; UA: uronic acid; Hex: hexose; 1S, a sulfate at position 1; 4S, a sulfate at position 4.

Supplementary Figure legend

Supplementary Fig S1. 3D structure of IDS and amino acid alignment of CXPXR motif in mammals. (A) 3D structure of human IDS (UniProt ID: P22304) with P86. PolyPhen-2, a prediction program of functional effects of human nsSNPs (http://genetics.bwh.harvard.edu/pph2/) was used. (B) Amino acid alignment of CXPXR motif and other amino acids which are predicted to be interacted with human IDS P86L. Query: Homo sapiens (human); sp|G1T2G7#1: Oryctolagus cuniculus (Rabbit); sp|G1M8Q2#1: Ailuropoda melanoleuca (Giant panda); sp|G1NSF9#1: Myotis lucifugus (Little brown bat); sp|UPI00022B6203#1: Cavia porcellus (Guinea pig); sp|UPI0000EBEC1D#1: Bos taurus (Bovine)/Bos mutus grunniens (Wild yak); sp|G3T583#1: Loxodonta africana (African elephant); sp|Q32KH3#1: Canis lupus familiaris (Dog) (Canis familiaris); sp|UPI0002236244#1: not active; sp|Q08890#1: Mus musculus (Mouse); sp|F7BPT3#1: Deleted; sp|Q3V1R8#1: Mus musculus (Mouse); sp|Q32KJ4#1: Rattus norvegicus (Rat); sp|UPI0001CF3E3C#1: not active; sp|F1N2D5#1: Bos taurus (Bovine).

Supplementary Fig S2. Genotyping of a novel *Ids*-P88L MPS II mouse model. (A) Specific probes for wild-type (FAM) and *Ids*-P88L (HEX) designed and used for quantitative PCR amplification. (B) Allelic discrimination plot of genotyping. A group of wild-type (blue), *Ids*-P88L (red), and heterozygote (*Ids*-P88L/wild-type) was presented.

Supplementary Fig S3. Quantification of MPS-specific biomarkers in visceral organs in Ids-P88L MPS II mouse model. (A) Representative chromatogram for HN-UA(1S) and HNAc-UA(1S) in the liver of wild-type and an *Ids*-P88L MPS II mouse model. Top, wild-type; bottom, *Ids*-P88L MPS II mouse model. (B) Quantitative results of HN-UA(1S) in the visceral organs. (C) Quantitative results of HNAc-UA(1S) in the visceral organs. The level of these biomarkers in the liver, kidney, spleen, lung, and heart was examined by LC-MS/MS. Data were expressed as relative amount after normalization using protein concentration (n = 4 – 6). Each circle represents an individual mouse. Open circle, wild-type, closed circle, *Ids*-P88L MPS II mouse model. A bar indicates mean value. * denotes $P < 0.05$. (D) Quantitative results of HNAc-UA(1S) in DBS. Data were expressed as relative amount after normalization using di-4S (n = 11 for WT; n = 13 for IDS-P88L MPS II mouse model).

Supplementary Fig S4. Graphical presentation of gene correction strategy for *Ids*-P88L MPS II mouse model. (A) The position of PAM was underlined. To improve gene correction efficiency, a C-to-G conversion (red) was induced by Cas9 nuclease, gRNA, and ssODN for template DNA. (B) Cas9-mediated DNA cleavage assay. A 615 bp DNA fragment (100 ng) was incubated with a complex of Cas9 protein (0.5 μg) and sgRNA (50 ng) in 50 mM Tris-HCl (pH 7.9) containing 100 mM NaCl, 10 mM $MgCl_2$ and 1 mM dithiothreitol at 37° C for 2 h. Reaction was separated using a 2.5% agarose-gel followed by ethidium bromide detection. Only a large DNA fragment (approximately 443 bp) was detected.

References

[1] R. Mashima, M. Ohira, T. Okuyama, A. Tatsumi, Quantification of the enzyme activities of iduronate-2-sulfatase, N-acetylgalactosamine-6-sulfatase and N-acetylgalactosamine-4-sulfatase using liquid chromatography-tandem mass spectrometry, Mol Genet Metab Rep. 14 (2018). https://doi.org/10.1016/j.ymgmr.2017.12.001.
[2] M. Ohira, T. Okuyama, R. Mashima, Quantification of 11 enzyme activities of lysosomal storage disorders using liquid chromatography-tandem mass spectrometry, Mol Genet Metab Rep. 17 (2018) 9–15. https://doi.org/10.1016/j.ymgmr.2018.08.005.
